# Gene expression analysis in allergology: the prediction of *Hymenoptera* venom allergy severity and treatment efficacy

**DOI:** 10.1186/2045-7022-3-35

**Published:** 2013-10-27

**Authors:** Marek Niedoszytko, Marta Gruchała-Niedoszytko, Ewa Jassem

**Affiliations:** 1 Department of Allergology, Medical University of Gdansk, Debinki 7, 80-210, Gdansk, Poland; 2 Department of Clinical Nutrition, Medical University of Gdansk, Debinki 7, 80-210, Gdansk, Poland

**Keywords:** Insect venom allergy, Gene expression, Immunotherapy, Mastocytosis

## Abstract

Insect venom allergy (IVA) may result in the most severe systemic reactions seen in allergology. The only potentially curative treatment option is venom immunotherapy (VIT) over 3 to 5 years. This treatment is effective in more than 90% of subjects but no reliable predictors of VIT effectiveness exist. Sting challenge with a living insect can be performed to assess the effectiveness of VIT: the predictive value of sting challenge can be highly sensitive in patients with honeybee venom allergy whereas in yellow jacket allergy, a negative result can be reliable if the challenge has been repeated at least 3 times.

The analysis of gene expression may be a step towards personalized venom immunotherapy assessing the effectiveness of treatment, the minimal required time for VIT and the persistence of long term tolerance induced by the treatment. Recent studies have enabled construction of a predictive model that could potentially be used in clinical practice to assess the efficacy of insect venom immunotherapy. A set of 69 genes that may be responsible for long-term protection was identified. Further analysis of the previously identified 6 transcripts make up the 18 gene predictive peripheral blood showed differences in patients treated with IVA. Further studies are needed to investigate the usefulness of gene expression analysis and other markers in the prediction of VIT effectiveness.

## What may genetic analysis add to allergology?

Genetic analysis has now become routine in several fields of medicine (e.g. oncology, haematology). The first example of the pharmacogenetic approach is the MammaPrint test analyzing the expression of 70 genes from an early-stage breast cancer tissue sample to figure out if the cancer has a low or high risk of recurrence within 10 years of diagnosis. As a result, the patients are grouped into 2 categories of recurrence risk: low, (less than 10% without additional treatment) and high (more than 29% without additional treatment). The test can prevent the development of side effects of chemotherapy in patients with a low risk of recurrence [1]. The analysis of *KIT* mutations in the diagnosis of systemic mastocytosis [2] and the PDGFRA/FIP1L1 fusion gene in hypereosinophilic syndrome [3] are rare examples of the application of pharmacogenetics in allergology. The current data do not support the widespread, everyday, clinical use of DNA or RNA analysis in other allergic diseases [4]. Research on gene expression in allergen immunotherapy is less developed by comparison with studies on asthma and oncology. The number of publications on gene expression and allergen immunotherapy gathered in PubMed database in July 2013 was only 126 (among them 20 on insect venom allergy). By comparison, gene expression was described in 3,302 publications on asthma and 15,734 on lung cancer. However it seems that it is only a matter of time before this situation changes.

The analysis of DNA describes the “anatomy” of our genome as polymorphisms and mutations, which may be responsible for differences in the phenotype. The anatomy of the gene is stable and depends on heredity. Somatic mutations are the exception, as they can occur at any moment of life. In general when we analyze the genome by PCR (polymerase chain reaction) or GWAS (genome-wide association study) the majority of the information (except somatic mutations) will be valid throughout the individual’s life. Gene expression looks at the way genes work. It can vary in the course of the disease and furthermore, can be modified by treatment. Thus single gene expression analysis and whole genome expression studies describe the function of the genome at the time of the analysis. Both studies on DNA and RNA may help to tailor the personalized treatment of the patient.

The data obtained from studies on whole genome expression or GWAS are large and difficult to analyze without the mathematical tools to support the diagnosis. Furthermore the large datasets of electronic medical records can be combined with the genetic data and help to establish the clinical decision [4].

## The unanswered questions in insect venom allergy

The only treatment addressing the cause of IVA is venom immunotherapy (VIT) for 3 to 5 years. In more than 95% of subjects the treatment is effective, as demonstrated by sting challenges or field stings. None of the *in vitro* methods used so far may replace the sting challenge and predict ineffectiveness of VIT. The data published in the Cochrane Review of insect venom immunotherapy did not identify any difference in the effectiveness of VIT in the compared patient groups or modes of immunotherapy [5]. The further part of the paper describes the components of the decision support tool, which could be used in the future to assess the effectiveness of VIT including clinical data, coexistence of mastocytosis, gene expression results, Tryptase levels and other *in vitro* markers.

## Gene expression

Recently data on gene expression has been analyzed for immunotherapy to grass, house dust mites and ovalbumin [6-9]. Our research group has focused on the patients with insect venom allergy [10-12]. Our aim was to define the gene expression pattern to assess the effect of the treatment with a less invasive method than the sting challenge and to assess the risk of the development of mastocytosis, which can predict the lack of long-term protection of VIT. There is still an unanswered question in allergology relating to the long-term protection achieved by immunotherapy [10,11]. Retrospective analysis in relation to field insect sting reactions can be an easy marker of the tolerance gained by the treatment. Our study on the prediction of the effectiveness of venom immunotherapy was performed in few patient groups: patients before venom immunotherapy, on the maintenance phase of the treatment, those treated with the VIT in the past who were stung and tolerated the venom, and those who did not benefit from VIT. The results of the study indicate that the effectiveness of VIT can be assessed with gene expression studies [10]. Differences in gene expression are related to the known mechanisms in differentiation of T lymphocytes, mast cell activation genes related to FcγR1, JAKSTAT, MAPK, Wnt, and calcium channels pathways. It also recognizes new genes such as TWIST-2, CLDN1 and PRLR as predictors of VIT effectiveness. TWIST-2 is a transcription factor stimulating IL-10 and decreasing IL-4 expression. CLDN 1 is an adhesion molecule, which enables the migration of dendritic cells and is related to the TGF-β level. However the transcripts of established function are only a proportion of the genes that show clinical relevance in differentiating between effectively and non – effectively treated patients. Most of the genes have an unknown function and the functional studies on their activity opens new field of research and let us realize how far we are from understanding the mechanisms of immunotherapy [10,11].

**Figure 1:**
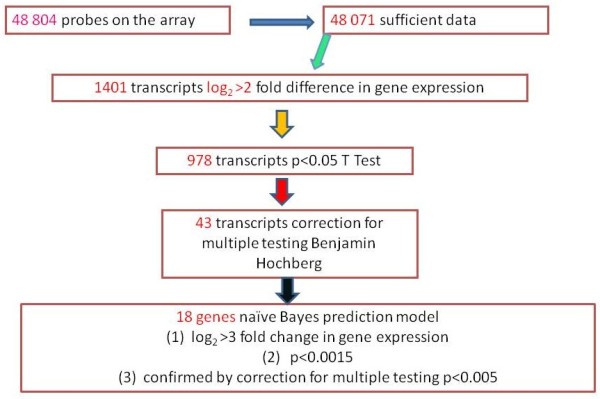
Development of the prediction model assessing the efficacy of VIT.

The gene expression profile indicated by the results of the study enables the construction of a prediction model, which could be used in clinical practice to assess the efficacy of insect venom immunotherapy. As a first step, patients who achieved venom tolerance were compared with subjects who did not tolerate the insect sting. The genes in the model were selected out of 48,804 probes on the array by the fold change in expression above 3, t-test p < 0.0015, Benjamin Hochberg correction for multiple testing p < 0.005. The set of 18 genes gave prediction results similar to the sets composed of a higher number of genes (Figure 1) [10].

**Figure 2:**
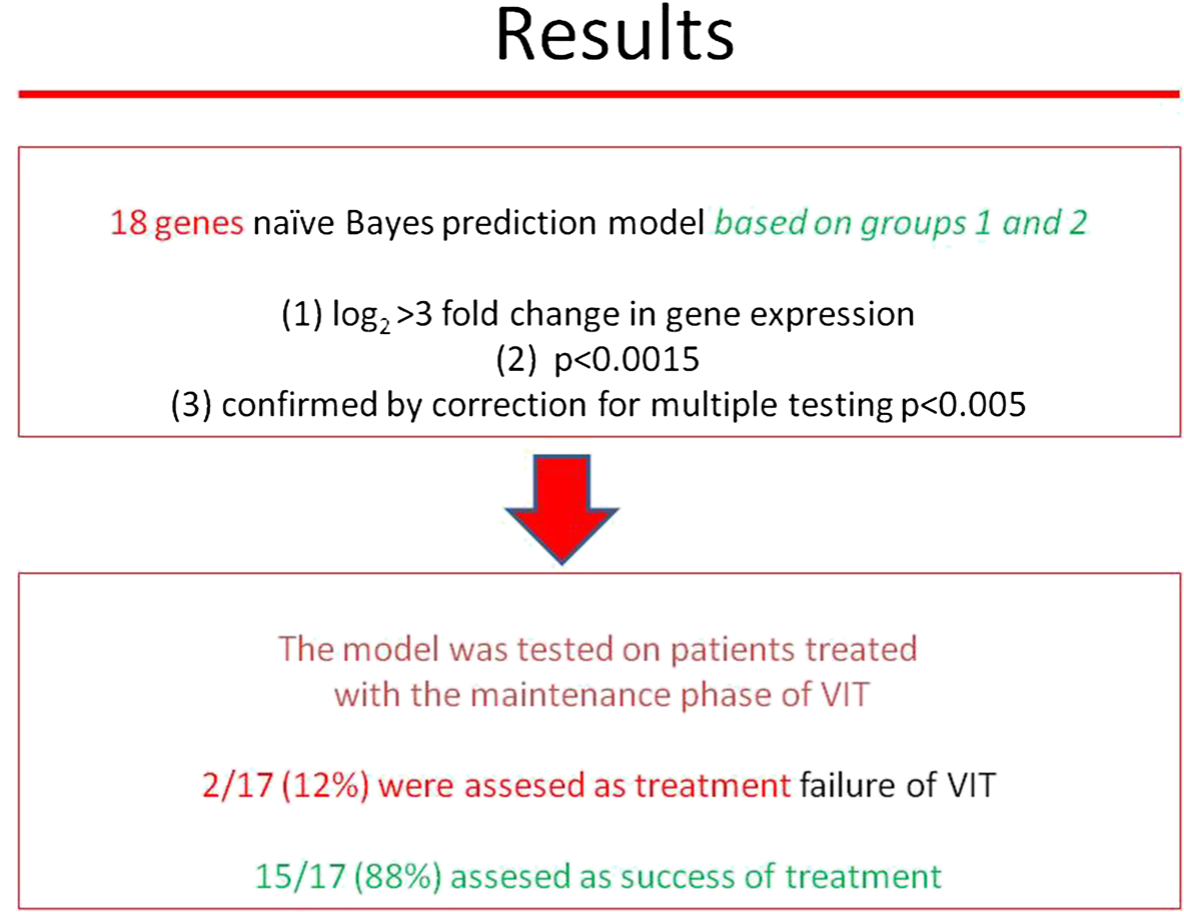
Use of the prediction model in the group of patients during the maintenance phase of VIT.

**Figure 3:**
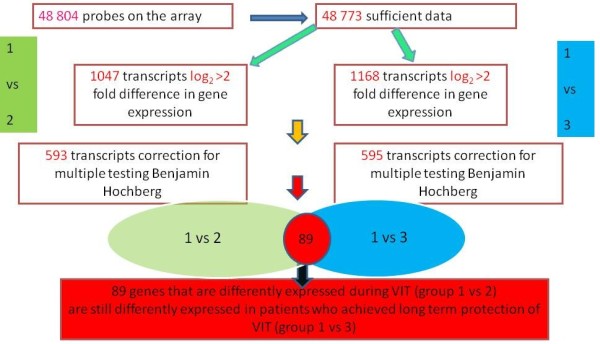
Differences in expression of the genes in the course of immunotherapy (patient group 1 – before immunotherapy, 2 – during the maintenance phase, 3 – patients who were treated in the past and achieved long term tolerance).

For the second stage, the gene model was tested on patients who were treated with venom immunotherapy predicting the effectiveness of VIT in 88% of treated subjects (Figure 2) [10]. Further analysis compared patients (groups 1 versus 2) before immunotherapy with the group on the maintenance phase of immunotherapy and patients before immunotherapy with those who were treated in the past and showed field sting tolerance (group 3) (Figure 3). As a result, a set of 89 genes that may be responsible for long term protection was identified. These genes are common in both comparisons, being differently expressed during the maintenance phase of VIT but still maintaining this pattern years after the end of VIT when long term tolerance is still protecting the patients. Furthermore when we analyzed these genes, we found among them six transcripts (C16ORF13, HS.583392, HS.532515, PRLR, TWIST2) [10], which compose the 18 genes prediction model of the effectiveness of VIT [11].

## Other components of the prediction model

The data published in the Cochrane Review, mentioned above, did not indicate any clinical marker that could predict the effectiveness of VIT [5]. Analysis of the retrospective data performed in patients treated with immunotherapy who also suffered from mastocytosis has shown impairment of effectiveness in comparison to the general population of treated subjects. However the risk benefit ratio favours VIT as untreated patients with mastocytosis with IVA are at risk of a fatal reaction [13]. The severity of the reaction to an insect sting is linearly correlated with the tryptase level [14]. The analysis of whole gene expression showed a difference in expression of both the mastocytosis patient who experiences an anaphylactic reaction to an insect sting in their medical history and the pattern of expression that can differentiate between a patient with mastocytosis and the general population [11,15].

Gene expression studies have also been performed in subjects with other allergic diseases. Gene expression was studied in a murine model of oral desensitization to ovalbumin, which showed differences in the intestinal epithelial cells [8]. A difference in gene expression was found in nasal mucosa as a result of pollen immunotherapy [7,16]. The improvement of molecular diagnostic methods in allergology has become one of the major scientific aims of the European Academy of Allergology and Clinical Immunology [17].

In summary, we can hypothesize the future development of a clinical tool, which can be used to address the currently unanswered question relating to the efficacy of insect venom immunotherapy, being created from clinical data, tryptase levels and gene expression studies dividing patients into 2 groups: those treated effectively and those not achieving clinical protection who require more intensive treatment.

## Abbreviations

VIT: Venom immunotherapy; IVA: Insect venom allergy; PCR: Polymerase chain reaction; GWAS: Genome-wide association study.

## Competing interests

The authors do not declare any competing interests related to the paper.

## Authors’ contributions

MN has made substantial contributions to conception, design and drafting of the manuscript; MGN participated in drafting of the manuscript; EJ has given final approval of the version to be published. All authors read and approved the final manuscript.
